# Cumulative Vulnerability in Cardiac Critical Care: A Framework for Understanding Healthcare-Associated Infections and Their Progression to Severe Infection and Sepsis

**DOI:** 10.3390/medicina62050908

**Published:** 2026-05-08

**Authors:** Daniela Mirela Vîrtosu, Angela Dragomir, Simina Crișan, Silvia Luca, Oana Pătru, Ruxandra-Maria Băghină, Mihai-Andrei Lazăr, Alina-Ramona Cozlac, Stela Iurciuc, Constantin Tudor Luca

**Affiliations:** 1 Doctoral School, “Victor Babes” University of Medicine and Pharmacy, 300041 Timisoara, Romania; daniela.cozma@umft.ro (D.M.V.); silvia.luca@umft.ro (S.L.); oana.patru@umft.ro (O.P.); ruxandra.croicu@umft.ro (R.-M.B.); 2 Institute of Cardiovascular Diseases Timisoara, 13A Gheorghe Adam Street, 300310 Timisoara, Romania; angela.dragomir@umft.ro (A.D.); lazar.mihai@umft.ro (M.-A.L.); alina-ramona.cozlac@umft.ro (A.-R.C.); constantin.luca@umft.ro (C.T.L.); 3 Research Center of the Institute of Cardiovascular Diseases Timisoara, 13A Gheorghe Adam Street, 300310 Timisoara, Romania; 4 Cardiology Department, “Victor Babes” University of Medicine and Pharmacy, 2 Eftimie Murgu Sq., 300041 Timisoara, Romania; iurciuc.stela@umft.ro

**Keywords:** healthcare-associated infections, coronary care unit, cumulative vulnerability, heart failure, invasive devices, left ventricular dysfunction, diabetes mellitus, infection prevention, sepsis, critical care

## Abstract

*Background and Objectives*: Healthcare-associated infections (HAIs) remain a relevant complication in coronary care units (CCUs), particularly among patients with cardiac dysfunction requiring invasive monitoring and prolonged hospitalization. In this setting, infection occurrence may reflect the cumulative interaction between baseline biological vulnerability and care-related exposure. This study aimed to explore whether a simple cumulative framework integrating these components can describe patterns of HAI occurrence and support early identification of patients at risk for severe infectious complications and sepsis. *Materials and Methods:* The retrospective cohort study included 870 consecutive adult patients admitted to a tertiary-care CCU. A four-component cumulative framework was constructed using reduced left ventricular ejection fraction (LVEF < 40%), diabetes mellitus, urinary catheterization, and CCU length of stay > 5 days. Each component contributed one point (range 0–4). HAIs were defined according to CDC/NHSN criteria and required microbiological confirmation. Associations between cumulative burden and infection occurrence were assessed using trend analysis and exploratory modeling. *Results*: HAI occurrence increased progressively across cumulative framework levels, demonstrating a stepwise pattern from low to higher vulnerability strata (*p* for trend < 0.001). A substantial proportion of infections clustered in patients with higher cumulative values, despite representing a minority of the cohort. Increasing cumulative burden was accompanied by higher observed infection occurrence, supporting a graded association between cumulative vulnerability and infection occurrence. *Conclusions*: In CCU patients, HAI occurrence appears to reflect the accumulation of biological vulnerability and care-related exposure during hospitalization. A simple cumulative framework may support early identification of patients requiring closer preventive attention and contribute to improved awareness of severe infectious complications in cardiac critical care. Prospective validation is warranted.

## 1. Introduction

Healthcare-associated infections (HAIs) remain an important cause of morbidity in coronary care units (CCUs), where patients are exposed to intensive monitoring, invasive procedures, and prolonged hospitalization [1]. Among individuals admitted to CCU, those with cardiac dysfunction and heart failure represent a particularly vulnerable population, characterized by hemodynamic instability, immune dysregulation, chronic inflammation, high device utilization, and frequent escalation of supportive therapies. In this setting, infection occurrence does not arise from a single determinant but rather from the interaction between patient-related vulnerability and cumulative exposure to hospital-related interventions [2,3]. HAIs in this population also represent an important precursor of severe infectious complications, including sepsis, which remains a major determinant of adverse outcomes in critically ill cardiac patients.

Cardiac dysfunction, particularly reduced left ventricular ejection fraction (LVEF), is associated with hemodynamic compromise that may influence tissue perfusion, especially in more severe cases, and may also contribute to systemic inflammatory activation and altered immune responsiveness. Patients with reduced LVEF often present with multiple comorbidities, metabolic disturbances, and neurohormonal dysregulation, all of which may compromise host defense mechanisms [4,5,6,7,8]. However, biological vulnerability alone is unlikely to fully explain infection occurrence in CCU patients [9]. Invasive devices such as urinary and central venous catheters, respiratory support, and extended length of stay represent well-established mediators of HAI development and tend to accumulate over time in patients with more severe cardiac disease [10,11,12,13].

Most existing approaches to HAI stratification have been developed in general intensive care settings and typically focus on isolated predictors, including device exposure, severity-of-illness scores, or comorbidity burden. Few approaches have specifically addressed the cardiac critical care population or attempted to integrate cardiac functional impairment with cumulative care-related exposure in a structured manner. As a result, clinicians lack a pragmatic framework that reflects the dynamic and progressive nature of infection occurrence in CCU patients [14,15,16].

Recent analyses of CCU cohorts suggest that reduced LVEF may function less as an independent causal determinant of infection and more as a marker of clinical vulnerability, identifying patients who are more likely to require invasive support and prolonged hospitalization. These observations support the hypothesis that infection occurrence in cardiac critical care is cumulative rather than static and emerges from the progressive interaction between baseline vulnerability and evolving hospital exposure [1,5,13,17,18].

The contribution of this approach lies not in the individual variables, which are well established, but in conceptualizing infection occurrence as a cumulative and dynamic process integrating baseline vulnerability with evolving care-related exposure.

Against this background, we aimed to develop an exploratory dynamic cumulative vulnerability framework tailored to the CCU population. This framework integrates baseline vulnerability markers, including reduced LVEF and diabetes mellitus, with care-related exposure variables such as urinary catheterization and prolonged CCU stay. The objective of this study was to evaluate whether a simple additive framework can describe patterns of HAI occurrence in CCU patients and reflect the cumulative nature of vulnerability to severe infectious complications.

## 2. Materials and Methods

This investigation was designed as a retrospective cohort study performed in the CCU of a tertiary referral center delivering advanced cardiovascular care to a large regional population in Western Romania. The study included all adult patients consecutively admitted to the CCU over a six-month interval, from 1 May to 31 October 2024.

During this period, 870 consecutive hospitalizations fulfilled the eligibility criteria and were retained for analysis. To preserve the real-world clinical spectrum of CCU practice, all patients aged 18 years or older were considered eligible regardless of their infection status at admission. Pre-existing infections documented at the time of admission were recorded as baseline clinical characteristics but were not categorized as HAIs. Exclusion criteria were restricted to the absence of an echocardiographic evaluation of left ventricular systolic function during hospitalization, lack of microbiological documentation, or incomplete clinical records that prevented reliable assessment of infectious outcomes.

LVEF was determined by transthoracic echocardiography carried out by experienced cardiologists using the biplane Simpson method. Whenever possible, the first available examination performed shortly after admission—typically within the initial 24 h of CCU hospitalization—was used for analysis, in accordance with contemporary echocardiographic practice guidelines. For analytical purposes, patients were categorized according to systolic function into those with reduced LVEF (<40%), indicating systolic dysfunction, and those with preserved or mildly reduced systolic function (≥40%).

HAIs were identified according to the criteria established by the Centers for Disease Control and Prevention and the National Healthcare Safety Network (CDC/NHSN). Infections were considered healthcare-associated when they were neither present nor incubating at admission and were diagnosed at least 48 h after hospital entry. Only microbiologically confirmed infections were included in the analysis. Confirmation relied on culture results obtained from relevant clinical specimens, including blood cultures, urine cultures, respiratory samples, wound or pressure-ulcer cultures, catheter-tip cultures, and assays detecting *Clostridioides difficile* toxins. Patients with colonization or infection at admission were not excluded from the cohort; these conditions were recorded as baseline characteristics but were not classified as HAIs. Microbial colonization detected during admission screening was recorded separately and was not treated as infection. In cases where infection occurred during hospitalization, microbiological findings were reviewed to distinguish new infections from pre-existing conditions. All patients underwent routine clinical monitoring during CCU stay. The timing of HAI diagnosis was reviewed in relation to invasive device exposure, and infections were observed to occur after the initiation of care-related exposures, including urinary catheterization when present.

Information used in this analysis was retrieved from electronic medical records and was limited to the CCU hospitalization episode. Extracted variables included demographic characteristics (age and sex), relevant comorbid conditions such as diabetes mellitus, admission characteristics, exposure to invasive devices including urinary catheterization, and length of stay in the CCU.

The study protocol was approved by the institutional ethics committee and conducted in accordance with the ethical principles outlined in the Declaration of Helsinki.

### 2.1. Selection of Variables for the Cumulative Framework

Variables included in the cumulative framework were selected a priori based on biological plausibility and clinical relevance in the CCU setting. The selection strategy was intentionally designed to avoid overfitting in a low-event setting and to prioritize interpretability and clinical applicability. Two conceptual domains were considered: baseline biological vulnerability and care-related cumulative exposure.

Baseline vulnerability was represented by reduced LVEF (LVEF < 40%) and the presence of diabetes mellitus. Reduced LVEF was selected as an indicator of systolic dysfunction and hemodynamic compromise, whereas diabetes mellitus was included as a marker of metabolic and immunological susceptibility commonly encountered in cardiac patients. These variables represent pre-existing patient-related vulnerabilities present at or shortly after admission.

Care-related exposure variables included the presence of a urinary catheter during CCU stay and a CCU length of stay (LOS) > 5 days. Urinary catheterization was selected as a marker of invasive procedural exposure and potential disruption of natural host barriers. Central venous catheterization was not included in the cumulative framework, as it was present in the vast majority of patients in this CCU cohort and therefore did not provide sufficient variability to contribute to risk stratification. In contrast to baseline vulnerability markers, LOS was treated as a time-dependent exposure variable reflecting cumulative contact with the hospital environment and invasive care, rather than as an independent baseline predictor. The potential bidirectional relationship between LOS and HAI was acknowledged, and the cumulative framework should not be interpreted as causal. The cutoff of LOS > 5 days was predefined based on cohort distribution and clinical relevance as an indicator of prolonged CCU hospitalization, rather than derived from outcome-based optimization. This threshold was selected to provide a simple and clinically interpretable marker of sustained exposure while maintaining analytical stability in a low-event setting. Patients were monitored daily, and the cumulative framework was reassessed throughout hospitalization. Infection timing was reviewed using clinical records to ensure that exposure variables preceded HAI identification. The selection of variables was further informed by prior analyses in a similar CCU cohort, where multivariable modeling identified markers of vulnerability associated with HAI occurrence, supporting the inclusion of clinically relevant and readily available parameters in the present cumulative framework [6]. To enhance bedside applicability, the number of variables was deliberately restricted to a small set of dichotomous components that are routinely available in clinical practice. All variables were coded dichotomously, with 0 indicating absence and 1 indicating presence.

### 2.2. Construction of the Dynamic Cumulative Framework

A simple additive framework was constructed by assigning one point to each selected variable when present. Equal weighting was deliberately chosen to prioritize interpretability and bedside applicability over statistical optimization. The resulting cumulative framework values ranged from 0 to 4 points and reflected the progressive accumulation of baseline biological vulnerability and care-related exposure factors.

For descriptive analysis, patients were grouped according to their total range value (0, 1, 2, and 3–4 points). The highest framework categories were combined in selected analyses because of the limited number of outcome events observed in the cohort.

The cumulative framework range was primarily analyzed as an ordinal variable to evaluate the relationship between increasing cumulative burden and HAI occurrence. In addition, a secondary dichotomous stratification was performed, separating patients into lower (0–1 point)/higher (≥2 points) categories. This threshold was selected pragmatically to reflect the presence of at least two vulnerability or exposure components and to improve analytical stability in a low-event setting. It was not derived from outcome-based optimization and should not be interpreted as a validated cutoff.

Because care-related exposure variables may arise during hospitalization, the cumulative framework may change over time as additional vulnerability components become present. Accordingly, the framework was conceptualized as a dynamic stratification approach reflecting the progressive accumulation of vulnerability and exposure during CCU hospitalization, rather than as an admission-based predictive model intended to guide immediate therapeutic decisions.

### 2.3. Statistical Analysis

All collected data were entered into a Microsoft Excel database, version 2011 (Microsoft Corp., Redmond, WA, USA). Statistical analyses were performed using MedCalc for Windows, version 19.4 (MedCalc Software, Ostend, Belgium) and the Epi Info statistical package, version 7.2.5.0 (Centers for Disease Control and Prevention, Atlanta, GA, USA). Continuous variables were summarized as median (interquartile range) or mean ± standard deviation, as appropriate. Categorical variables were expressed as absolute frequencies and percentages. HAI occurrence was calculated across cumulative framework categories (0–4). Differences in infection proportions between framework categories were initially evaluated using the chi-square test. In addition, a chi-square test for trend was applied to assess the presence of a linear increase in HAI occurrence across the ordered framework categories.

To explore the patterns of association between cumulative vulnerability and infection occurrence, logistic regression analysis was applied in an exploratory manner, with HAI as the dependent variable and the cumulative framework entered as an ordinal variable. Odds ratios (ORs) with 95% confidence intervals (CIs) were estimated per one-point increase in framework level; however, these estimates were not intended to provide stable or generalizable effect sizes given the limited number of events. For that reason, an exact approach was additionally used. Specifically, Fisher’s exact test for trend was performed to assess the association between cumulative framework levels and HAI occurrence in a model-independent manner.

For clinically interpretable stratification, the cumulative framework was further dichotomized into lower (0–1) and higher (≥2) categories. This categorization was selected pragmatically to improve interpretability and analytical stability and was not derived from outcome optimization.

Given the limited number of outcome events (*n* = 16), multivariable adjustment was not considered statistically appropriate due to events-per-variable constraints and the risk of model instability and overfitting. The analyses were therefore intentionally restricted to exploratory stratification and pattern assessment rather than development of a multivariable predictive model.

A two-sided *p*-value < 0.05 was considered statistically significant.

## 3. Results

### 3.1. Cohort Characteristics

The study cohort included 870 consecutive CCU patients. During hospitalization, 16 HAIs were identified, corresponding to an overall incidence of 1.8%. A detailed distribution of HAI types was as follows: bloodstream infections (*n* = 7), urinary tract infections (*n* = 5), Clostridioides difficile infections (*n* = 2), one respiratory infection, and one surgical site infection related to device implantation. Bloodstream infections represented the most frequent type, consistent with the high use of invasive vascular access in the CCU setting.

No additional patient exclusions occurred for the present analysis, and complete data were available for all variables included in the cumulative framework.

Baseline characteristics stratified according to cumulative framework category are presented in Table 1.

### 3.2. Distribution of the Dynamic Cumulative Framework

The cumulative framework ranged from 0 to 4. Its distribution in the overall cohort was as follows: 325 patients (37.4%) had a value of 0, 242 (27.8%) had a value of 1, 178 (20.5%) had a value of 2, 100 (11.5%) had a value of 3, and 25 (2.9%) had a value of 4.

The framework showed a broadly progressive distribution without pronounced clustering at the extremes. In total, 125 patients (14.4%) had values ≥ 3, representing the subgroup with the highest cumulative vulnerability.

### 3.3. HAI Incidence Across Framework Categories

HAI incidence increased progressively across cumulative framework categories. No infections occurred among patients with a value of 0. Among those with values of 1, 2, 3, and 4, the observed infection rates were 1.24%, 2.81%, 6.00%, and 8.00%, respectively.

This pattern indicates a stepwise increase in infection occurrence across ordered framework categories. Notably, 8 of the 16 HAIs (50%) occurred in patients with values ≥ 3, although this subgroup represented only 14.4% of the total cohort, indicating a concentration of events at higher cumulative levels. Confidence intervals were wider in the highest category, reflecting the limited number of outcome events.

A chi-square test for trend indicated a statistically significant increase in HAI incidence across framework categories (*p* < 0.001). The association remained significant when assessed using exact methods (Fisher’s exact test for trend, *p* < 0.001), supporting the robustness of the observed monotonic pattern. The distribution of HAIs by cumulative framework category is summarized in Table 2.

**Figure 1:**
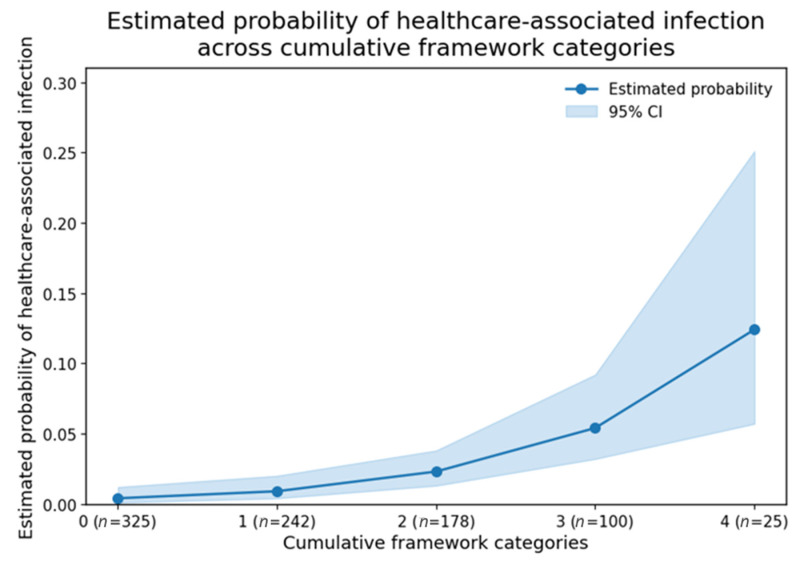
Estimated probability of HAI across cumulative framework categories. Estimated probabilities derived from exploratory logistic regression illustrate an increasing pattern in infection occurrence across cumulative framework categories. Shaded areas represent 95% CI. Estimates are based on exploratory modeling and may be unstable given the limited number of events; visual interpretation of effect size magnitude should therefore be approached with caution.

Estimated probabilities from exploratory logistic regression showed a similar increasing pattern (Figure 1). However, given the limited number of outcome events, these model-based estimates should be interpreted with caution, as they may be affected by small-sample instability.

### 3.4. Dichotomized Framework Analysis

For clinically interpretable stratification, the cumulative framework was dichotomized into lower (0–1) and higher (≥2) categories.

The lower category comprised 567 patients, among whom 3 HAIs were recorded (0.53%). The higher category included 303 patients, within which 13 HAIs occurred (4.29%), corresponding to an absolute difference of 3.76 percentage points between categories. This descriptive comparison indicates a higher occurrence of HAIs in patients with cumulative framework values ≥ 2.

An exploratory logistic regression analysis was accompanied by higher estimated odds of HAI occurrence in the higher category compared with the lower category (OR 8.43, 95% CI 2.38–29.81; *p* < 0.001). However, given the limited number of outcome events (*n* = 16), this estimate is likely unstable and may overestimate the magnitude of association, and should therefore be interpreted with caution.

### 3.5. Ordinal Logistic Analysis

When the cumulative framework was entered as an ordinal predictor in logistic regression analysis, each one-point increase was associated with higher estimated odds of HAI occurrence (OR 2.48; 95% CI 1.61–3.82; *p* < 0.001). However, given the limited number of outcome events (*n* = 16), this model-based estimate is likely unstable and may overestimate the magnitude of association.

**Figure 2:**
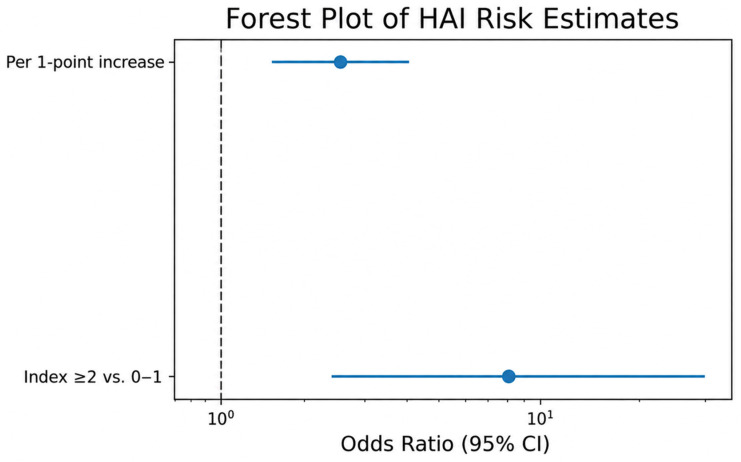
ORs for HAIs according to the cumulative framework. ORs and 95% CIs derived from exploratory logistic regression are shown for higher (≥2 vs. 0–1) framework categories and per one-point increase in the cumulative framework. The vertical dashed line indicates the null value (OR = 1). Estimates are based on exploratory modeling and may be unstable given the limited number of events; visual interpretation of effect size magnitude should therefore be approached with caution.

Accordingly, the ordinal logistic analysis should be regarded as exploratory and interpreted only as supportive of the descriptive increase in infection occurrence observed across cumulative framework categories, rather than as a reliable measure of effect size. The magnitude of association for both ordinal and dichotomized analyses is illustrated in Figure 2, and should be interpreted with caution.

Overall, the cumulative framework showed consistent patterns across descriptive, dichotomized, and ordinal analyses.

## 4. Discussion

### 4.1. Principal Findings

In this exploratory analysis of a CCU cohort, we observed a progressive increase in HAI occurrence across cumulative framework levels. Infection incidence ranged from 0% among patients with a value of 0 to 8% among those with a value of 4. Notably, half of all HAIs occurred in patients with values ≥ 3, although this subgroup represented a relatively small proportion of the overall cohort. Exploratory model-based analysis suggested a similar pattern; however, given the limited number of events, these estimates are likely unstable and should not be interpreted as reliable measures of effect magnitude.

Overall, these findings suggest that infection occurrence in cardiac critical care may reflect the accumulation of biological vulnerability and care-related exposure during hospitalization, rather than being determined by a single factor.

In a previous analysis of this cohort, malignancy was associated with HAI occurrence in an exploratory multivariable model [6]. However, its relatively low prevalence and limited distribution within the CCU population reduce its practical utility when constructing a cumulative bedside stratification framework. In contrast, diabetes mellitus represents a more prevalent and clinically relevant marker of systemic vulnerability in cardiac patients and was therefore incorporated into the present framework. Together, these observations support the concept that infection susceptibility in CCU patients may arise from the interaction between baseline vulnerability and cumulative exposure to invasive care.

### 4.2. Pathophysiological Interpretation

The cumulative structure of the proposed framework is consistent with biologically plausible mechanisms for infection development in CCU patients. Reduced LVEF is associated with impaired tissue perfusion, neurohormonal activation, endothelial dysfunction, and altered immune responsiveness—factors that may influence susceptibility to infectious complications [4,19,20]. Diabetes mellitus contributes additional metabolic and microvascular vulnerability [21,22,23]. Together, these factors represent baseline biological susceptibility present early during hospitalization.

However, baseline vulnerability alone is unlikely to fully account for infection occurrence. Care-related exposures, particularly invasive devices and prolonged hospitalization, create opportunities for microbial entry and increase the duration of contact with the hospital environment [2]. Urinary catheterization, for example, directly disrupts natural host barriers [24,25,26], whereas longer CCU stays increase cumulative exposure to invasive procedures and healthcare-associated pathogens [1,2,27,28].

The progressive increase in HAI incidence across cumulative framework levels is consistent with the hypothesis that infection occurrence in cardiac critical care may arise from the interaction between patient-related vulnerability and the accumulating burden of procedural exposure. Within this context, infection susceptibility may be understood as an evolving, exposure-dependent process rather than a static phenomenon based solely on admission characteristics.

### 4.3. Methodological Rationale for the LOS Cutoff

The choice of a CCU LOS threshold of >5 days was based on both clinical reasoning and the distribution of hospitalization duration within the cohort. Prolonged hospitalization is a well-recognized exposure-related factor associated with HAIs, reflecting cumulative contact with the hospital environment, repeated procedural interventions, and increased opportunities for microbial colonization.

Within the present cohort, 5 days represented a practical threshold separating short-term stabilization from more prolonged critical care management. Patients exceeding this duration were more likely to require sustained invasive monitoring, continued device use, and ongoing therapeutic adjustments, all of which may contribute to increased exposure to infectious complications. Importantly, this threshold was defined a priori and was not derived from outcome-driven optimization procedures. The intention was to maintain interpretability and clinical applicability rather than to maximize statistical discrimination.

LOS should therefore be interpreted as a time-dependent exposure marker reflecting cumulative contact with the hospital environment, rather than as an independent causal determinant of infection occurrence. A bidirectional relationship between prolonged hospitalization and infection cannot be excluded, as the development of infection may itself contribute to extended LOS.

Future studies incorporating time-dependent analyses or alternative exposure metrics, such as device-days or continuous modeling of hospitalization duration, may further refine the understanding of temporal relationships between hospitalization and infection occurrence. Nevertheless, within the context of this cumulative framework, the >5-day cutoff provides a simple and clinically interpretable marker of sustained exposure burden rather than a validated predictive threshold.

### 4.4. Comparison with Existing Literature

Stratification approaches for HAIs have been widely investigated in general ICU populations, most commonly focusing on isolated predictors such as device exposure, severity-of-illness scores (e.g., APACHE or SOFA), or comorbidity burden. Although these approaches provide important epidemiological insight, they are typically derived from heterogeneous ICU populations and may not fully reflect the specific clinical context of cardiac critical care [1,14,16,29,30,31].

CCUs differ from general ICUs in several respects, including the high prevalence of myocardial dysfunction, continuous rhythm monitoring, and frequent use of invasive diagnostic and therapeutic procedures. In this setting, cardiac functional impairment represents an important component of physiological reserve and may influence susceptibility to infectious complications during hospitalization [32,33,34].

In a previous analysis of this cohort, reduced LVEF was associated with HAI occurrence; however, this association was attenuated after adjustment for exposure-related variables [6]. This suggests that cardiac dysfunction may function less as an independent determinant and more as a marker of vulnerability associated with increased exposure to invasive care.

The present study builds on these observations by integrating cardiac dysfunction within a cumulative framework. Rather than evaluating individual variables in isolation, this approach conceptualizes infection occurrence as the combined effect of baseline vulnerability and care-related exposure, evolving over time.

Although some ICU-based models incorporate comorbidity indices or counts of invasive devices, relatively few have explicitly incorporated cardiac functional status within a cumulative stratification framework tailored to the CCU population [16,35,36]. This analysis therefore provides a CCU-focused perspective on infection occurrence, emphasizing the interaction between physiological reserve and procedural exposure.

### 4.5. Clinical Implications and Implementation Framework

The cumulative framework proposed in the present study was not designed as an admission-based predictive algorithm, but rather as an exploratory stratification approach that may support awareness of infection prevention needs throughout CCU hospitalization.

In contrast to static admission-based models, this framework reflects the progressive accumulation of vulnerability and exposure. Baseline factors such as reduced LVEF and diabetes mellitus are identifiable early, whereas care-related exposures, including device use and length of stay, evolve during hospitalization. This dynamic structure allows the framework to be updated over time, aligning stratification with the temporal nature of HAI development. From a practical perspective, the framework could be implemented as a simple four-component checklist integrated into routine clinical workflows. Because all variables are readily available during standard CCU care, its use would not require additional testing or resources.

Within this context, the framework may help identify patients in whom closer attention to device management and infection prevention practices is warranted. Patients with higher cumulative values may benefit from increased vigilance and more frequent reassessment of device necessity. The framework also aligns with the concept of device stewardship, providing a structured prompt to reassess ongoing invasive support. In addition, it may facilitate communication within the multidisciplinary team by enhancing awareness of cumulative vulnerability during daily care.

These potential applications remain conceptual. The present study does not demonstrate that framework-guided interventions reduce HAI incidence, but rather provides a structured approach to identifying patients at increased risk. Prospective studies are required to determine whether integrating cumulative stratification into routine practice improves infection-related outcomes. In this context, HAIs may represent an upstream step in the progression toward more severe infectious complications, including sepsis, particularly in vulnerable CCU populations.

### 4.6. Conceptual Shift from Predictive Modeling to Cumulative Stratification

A central conceptual element of the present study is the distinction between predictive modeling and cumulative stratification. Traditional predictive models aim to estimate individual outcome probabilities using multivariable techniques that require large numbers of events and rigorous validation. In low-event settings, such approaches may become unstable and prone to overfitting [30,36,37,38,39].

In contrast, the cumulative framework proposed here was not intended as a predictive model, but as a structured stratification approach reflecting the accumulation of vulnerability and exposure-related factors. The aim is not to generate precise individualized risk estimates, but to identify patterns of increasing vulnerability associated with infection occurrence. This approach has several implications. It acknowledges the statistical constraints imposed by the limited number of events and avoids reliance on potentially unstable model estimates. At the same time, it reflects the dynamic nature of infection occurrence in CCU patients, allowing stratification to evolve as exposure-related factors accumulate during hospitalization. In addition, the simplicity of the additive structure enhances clinical usability. Unlike more complex predictive models, the framework can be readily applied at the bedside without computational support, facilitating its potential integration into routine practice.

Finally, the framework should be viewed as hypothesis-generating. By identifying patterns of cumulative vulnerability, it provides a conceptual basis for the future development and validation of more refined predictive models in larger CCU populations.

### 4.7. Limitations

Several limitations should be acknowledged when interpreting the present findings. First, the relatively small number of HAI events (n = 16) limits the precision of effect estimates and precludes more complex modeling or validation procedures. Model-based estimates, including odds ratios, may therefore be unstable and should be interpreted with caution. Accordingly, the analysis should be considered exploratory and hypothesis-generating rather than confirmatory.

Second, the retrospective observational design introduces the possibility of residual confounding. Although the variables included in the cumulative framework were predefined and routinely collected, additional factors such as severity-of-illness indices, nutritional status, or prior antimicrobial exposure were not captured and may influence infection susceptibility.

Third, the inclusion of LOS introduces potential reverse causality that may affect the internal validity of the framework, as infection occurrence itself may contribute to prolonged hospitalization. Therefore, LOS should be interpreted strictly as a marker of cumulative exposure rather than an independent determinant, and the observed associations should not be interpreted causally.

Fourth, exposure variables were simplified. Urinary catheterization was recorded as a dichotomous variable without accounting for timing or duration, and the framework does not fully capture the temporal complexity of device-related risk. Fifth, the study was conducted in a single tertiary-care CCU without external validation. Local clinical practices may therefore influence generalizability.

Finally, the framework components and thresholds were selected based on clinical reasoning and interpretability rather than statistical optimization, and therefore require external validation in independent cohorts. The proposed framework should be regarded as conceptual. It does not demonstrate that framework-guided interventions reduce HAI incidence, but rather provides a structured approach to identifying patients who may warrant increased preventive attention. Whether integration into routine clinical workflows translates into improved outcomes will require prospective evaluation in larger populations.

## 5. Conclusions

HAI occurrence in CCU patients may reflect the cumulative interaction between baseline biological vulnerability and progressive care-related exposure during hospitalization. In this cohort, a simple four-component cumulative framework integrating reduced LVEF, diabetes mellitus, urinary catheterization, and prolonged CCU stay was associated with a graded pattern of infection occurrence.

These findings support the relevance of cumulative vulnerability in patients with cardiac dysfunction requiring cardiac critical care, in whom biological susceptibility and procedural exposure frequently coexist. Within this context, infection occurrence may evolve dynamically during hospitalization rather than being determined solely by admission characteristics.

The proposed framework should be interpreted as an exploratory stratification approach rather than a validated predictive model. Its simplicity and clinical interpretability may support structured infection prevention efforts and help identify patients in whom increased attention to preventive practices may be warranted. Prospective validation in independent cohorts will be required to determine whether integrating cumulative stratification into routine clinical workflows is associated with improved infection-related outcomes.

## Figures and Tables

**Table 1:** Patient characteristics and exposure variables according to cumulative framework categories. Patients were stratified into lower (0–1) and higher (≥2) categories based on the cumulative framework. Continuous variables are presented as mean ± standard deviation or median [interquartile range], and categorical variables as number (%).

Variable	Lower (0–1) (*n* = 567)	Higher (≥2) (*n* = 303)
Age, years (mean ± SD)	63.0 ± 13.2	67.0 ± 11.9
Male sex, n (%)	378 (66.7%)	200 (66.0%)
LVEF (%), mean ± SD	46.4 ± 7.6	35.7 ± 11.0
LVEF < 40%, n (%)	54 (9.5%)	181 (59.7%)
Diabetes mellitus, n (%)	63 (11.1%)	126 (41.6%)
Urinary catheter, n (%)	72 (12.7%)	232 (76.6%)
LOS, days (median [IQR])	4 [2–5]	7 [5–10]
HAI occurrence, n (%)	3 (0.53%)	13 (4.29%)

**Table 2:** Incidence of HAIs across cumulative framework categories. The table summarizes the distribution of patients and the corresponding incidence of HAIs within each cumulative framework category.

Cumulative Framework	Total Patients (*n*)	HAI (*n*)	HAI (%)
0	325	0	0.0
1	242	3	1.24
2	178	5	2.81
3	100	6	6.00
4	25	2	8.00
Total	870	16	1.8

## Data Availability

The original contributions presented in this study are included in the article. Further inquiries can be directed to the corresponding authors.

## Abbreviations

The following abbreviations are used in this manuscript:

AUC	Area Under the Receiver Operating Characteristic Curve
CCU	Coronary Care Unit
EPV	Events per Variable
HAI	Healthcare-Associated Infection
ICU	Intensive Care Unit
LOS	Length of Stay
LVEF	Left Ventricular Ejection Fraction
LASSO	Least Absolute Shrinkage and Selection Operator
ROC	Receiver Operating Characteristic
TRIPOD	Transparent Reporting of a Multivariable Prediction Model for Individual Prognosis or Diagnosis
